# Higher Frequency of T-Cell Response to *M. tuberculosis* Latency Antigen Rv2628 at the Site of Active Tuberculosis Disease than in Peripheral Blood

**DOI:** 10.1371/journal.pone.0027539

**Published:** 2011-11-10

**Authors:** Teresa Chiacchio, Elisa Petruccioli, Valentina Vanini, Ornella Butera, Gilda Cuzzi, Linda Petrone, Giuseppe Matteucci, Francesco Nicola Lauria, Kees L. M. C. Franken, Enrico Girardi, Tom H. M. Ottenhoff, Delia Goletti

**Affiliations:** 1 Translational Research Unit, Department of Epidemiology and Preclinical Research, “L. Spallanzani” National Institute for Infectious Diseases (INMI), IRCCS, Rome, Italy; 2 Clinical Department, INMI, Rome, Italy; 3 Department of Infectious Diseases, Leiden University Medical Center, Leiden, The Netherlands; 4 Clinical Epidemiology Unit, Department of Epidemiology and Preclinical Research, INMI, Rome, Italy; McGill University, Canada

## Abstract

**Rationale:**

Due to the invasive nature of the procedures involved, most studies of *Mycobacterium tuberculosis* (*Mtb)*-specific immunity in humans have focused on the periphery rather than the site of active infection, the lung. Recently, antigens associated with Mtb-latency and -dormancy have been described using peripheral blood (PB) cells; however their response in the lung is unknown. The objective of this report was to evaluate, in patients prospectively enrolled with suspected active tuberculosis (TB), whether the latency antigen Rv2628 induces local-specific immune response in bronchoalveolar lavage (BAL) cells compared to PB cells.

**Material/Methods:**

Among the 41 subjects enrolled, 20 resulted with active TB. Among the 21 without active disease, 9 were defined as subjects with latent TB-infection (LTBI) [Quantiferon TB Gold In-tube positive]. Cytokine responses to Rv2628 were evaluated by enzyme linked immunospot (ELISPOT) assay and flow cytometric (FACS) analysis. RD1-secreted antigen stimulation was used as control.

**Results:**

There was a significantly higher frequency of Rv2628- and RD1-specific CD4^+^ T-cells in the BAL of active TB patients than in PB. However the trend of the response to Rv2628 in subjects with LTBI was higher than in active TB in both PB and BAL, although this difference was not significant. In active TB, Rv2628 and RD1 induced a cytokine-response profile mainly consisting of interferon (IFN)-γ-single-positive over double-IFN-γ/interleukin (IL)-2 T-cells in both PB and BAL. Finally, BAL-specific CD4^+^ T-cells were mostly effector memory (EM), while peripheral T-cell phenotypes were distributed among naïve, central memory and terminally differentiated effector memory T-cells.

**Conclusions:**

In this observational study, we show that there is a high frequency of specific T-cells for *Mtb-*latency and RD1-secreted antigens (mostly IFN-γ-single-positive specific T-cells with an EM phenotype) in the BAL of active TB patients. These data may be important for better understanding the pathogenesis of TB in the lung.

## Introduction

Tuberculosis (TB) is commonly thought to have a simple binary distribution, active disease and latent infection. Although latent TB infection (LTBI) is generally associated with bacterial containment in some inactive form in the granuloma [1], the current definition of LTBI includes a diverse range of individuals, from those who have completely cleared, to those who are incubating actively replicating bacteria in the absence of clinical symptoms [2], [3]. On the other side, active TB in humans and non-primate animals is characterized by diverse pathological presentations, ranging from sterile tissue to caseous hypoxic lesions containing variable numbers of bacteria, to liquefied cavities with a massive load of replicating organisms [2], [4]. Based on this, *M. tuberculosis* (Mtb) infection may therefore be better viewed as a continuous spectrum ranging from sterilizing immunity, to subclinical active disease, to fulminant active disease with conventional designations of LTBI, and active disease corresponding to partially overlapping regions of biological heterogeneity [4], [5].

Due to the invasive nature of the procedures involved, most studies of *Mtb*-specific immunity in humans have focused on peripheral blood (PB) rather than the site of active infection, the lung [1], [6]–[9]. However, tissue-specific mucosal immunity in the lung, as measured e.g. in bronchoalveolar lavage (BAL), may differ significantly from the periphery and may yield more relevant clues about the mechanisms and immune components of protection and disease.

During LTBI, the tubercle bacilli contained within granulomas [1] are thought to be subjected to nutrient and oxygen deprivation [10], [11]. As part of the *Mtb*-adaptive response to hypoxia, expression of the DosR regulon is observed. The functions of most DosR-regulon-encoded proteins, hereafter referred to as latency antigens, are still mostly unknown [12], [13]. Recently, we described that interferon (IFN)-γ responses to *Mtb* latency antigens Rv2628c [14] are associated with LTBI and that within the CD4^+^ T-cells the response to Rv3133c, Rv1733c and Rv2029c is mainly associated with cytokine mono-functional expression [15]-[18]. It would be important to evaluate the immune response to these antigens at the site of TB because as reported above, not all lesions in the human lung are active during the disease [4], [5]. Thus these latency antigen-specific T-cells may be present in the TB-affected lung in association with silent lesions, but this has not been evaluated yet.

Based on the expression of surface markers associated with cell maturation [19], CD4^+^ T-cells can be phenotypically divided into at least four different populations: naïve (N) defined as CD62L^+^, CD45R0^-^, central memory (CM) defined as CD62L^+^, CD45R0^+^, effector memory (EM) defined as CD62L^-^, CD45R0^+^ and terminally differentiated effector memory T-cells (EF) defined as CD62L^-^, CD45R0^-^. In several infection models including TB, it has been shown that effector T-cells are expanded during active replication, whereas only memory cells are detectable after control or eradication [20]-[22]. However, no characterization of the phenotype of *Mtb*-specific cells during active disease simultaneously evaluated in blood and BAL has been performed yet.

It has recently been shown that the response to RD1 [early secreted antigenic target (ESAT)-6 and culture filtrate protein (CFP)-10] in BAL is associated with active TB [23]–[25]. However, no characterization of the surface cell profile associated with cell maturation after specific-Mtb stimulation has been performed until now.

Millington *et al*. have shown a predominant profile of IFN-γ-secreting T-cells in the PB of active TB patients, whereas interleukin (IL)-2-secreting cells appear in patients after successful treatment and may be considered as a consequence of central memory T-cells expansion, caused by the reduced Mtb antigen load [26]. Similarly Biselli *et al* have shown that IL-2 secretion is associated to LTBI after long-term stimulation with RD1 antigens [27]. All together, these data indicate that the concomitant evaluation of IFN-γ and IL-2 may be instrumental in assessing the different stages of TB.

Therefore, we prospectively enrolled patients with a high suspect of active TB who were undergoing BAL, and used the enzyme-linked immunospot test (ELISPOT) to investigate whether the latency antigen Rv2628 induces local-specific immune response at the site of infection. Responses to RD1 antigens were evaluated as control [23], [28]. Furthermore, we analyzed the cytokines (IFN-γ and IL-2) produced and the phenotype of responding cells after specific antigen stimulation by flow cytometry (FACS).

## Results

### Characteristics of the population

Forty-one subjects with suspected active pulmonary TB disease, who had negative results in acid fast bacilli (AFB) smears from sputa, and who had consequently undergone BAL procedure for diagnostic purposes were enrolled. After enrollment, the diagnosis was microbiologically confirmed in 10 and based on clinical criteria in 10. Among the 21 subjects without active TB, 9 were defined as LTBI because they tested positive to QuantiFERON TB Gold-In tube (QFT-IT) (Cellestis Limited, Carnegie, Victoria, Australia). Demographic characteristics, Bacillus Calmette et Guérin (BCG) vaccination status, QFT-IT results and final diagnosis are reported in Table 1.

**Table 1:** Demographic and clinical characteristics of the subjects enrolled in the study.

	Active TB	No active TB		Total	p value
		With LTBI	No LTBI		
	N (%)				
	20 (48.8)	9 (22.0)	12 (29.3)	41(100.0)	
**Median Age (IQR)**	33.5 (27.7–44.5)	46.0 (32.50–63.50)	37.5 (30.5–57.2)	36.0 (29.5–52.5)	**0.185**
**Female gender**	8 (40.0)	5 (55.6)	4 (33.3)	17 (41.5)	**0.324**
**Origin**					**0.986**
Eastern Europe	12 (60.0)	3 (33.3)	5 (41.7)	20 (48.8)	
Western Europe	4 (20.0)	4 (44.4)	5 (41.7)	13 (31.7)	
Asia	3 (15.0)	1 (11.1)	-	4 (9.8)	
Africa	1 (5.0)	1 (11.1)	2 (16.7)	4 (9.8)	
**BCG**					**0.915**
Vaccinated	14 (70.0)	4 (44.4)	6 (50.0)	24 (58.5)	
Unvaccinated	6 (30.0)	5 (55.6)	6 (50.0)	17 (41.5)	
**QTF-IT**					**<0.0001**
Positive	17 (85.0)	9 (100.0)	-	26 (63.4)	
Negative	3 (15.0)	-	12 (100.0)	15 (36.6)	
**TB Diagnoses**					**NA**
Microbiological	10 (50.0)	-	-	-	
Clinical	10 (50.0)	-	-	-	
**Lung Diseases other than TB**					**NA**
Pneumonia	-	6 (66.7)	7 (58.3)	-	
Bronchitis and lung infiltrates	-	1 (11.1)	1 (8.3)	-	
Pleural effusion	-	1 (11.1)	1 (8.3)	-	
Lung Abscess	-	-	1 (8.3)	-	
Emphysema and lung infiltrates	-	1 (11.1)	2 (16.7)	-	

**Footnotes:** TB: Tuberculosis; IQR: Interquartile range; BCG: Bacillus Calmette et Guérin; QTF-IT: QuantiFERON TB Gold in Tube; NA: not available.

### Comparison of Rv2628- and RD1-induced cytokines responses in circulating and BAL lymphocytes by ELISPOT

*In vitro* analyses could be performed in most, but not all BAL samples, due to cell constraints or due to being scored as indeterminate or anergic (Table 2). Regarding the 12 patients with lung diseases other than TB, although the majority was analyzable (Table 2), no response to Rv2628 or RD1 was detected (data not shown) and therefore these data were not included in the analysis reported below.

**Table 2:** BAL samples obtained and analyzed in the enrolled patients.

	Active TB				LTBI				No LTBI			
	Samples not available for the analysis			Total samples analyzed	Samples not available for the analysis			Total samples analyzed	Samples not available for the analysis			Total samples analyzed
	Cell constraint	Cell anergy	Indeterminate		Cell constraint	Cell anergy	Indeterminate		Cell constraint	Cell anergy	Indeterminate	
**ELISPOT: N/total (%)**	-	1/20 (5)	3/20 (15)	16/20 (80)	-	1/9 (11)	1/9 (11)	7/9 (78)	-	-	3/12 (25)	9/12 (75)
**FACS: N/total (%)**	6/20 (30)	-	-	14/20 (70)	4/9 (44)	-	-	5/9 (56)	3/12 (25)	-	-	9/12 (75)

**Footnote:** ELISPOT: enzyme-linked immunosorbent spot; FACS: flow cytometric analysis; TB: tuberculosis; LTBI: latent tuberculosis infection; cell constraint: mononuclear cells from BAL < 6×10^6^; cell anergy: by ELISPOT, if the results are less than 20 SFC/well in the positive control; cell anergy: by FACS, if the results are less than 0.06% in the positive control; indeterminate: by ELISPOT, if the results are more than 10 SFC/well in the negative control.

We first used ELISPOT to analyze the response to Rv2628- and RD1-antigens in active TB and LTBI subjects separately, comparing BAL cells (BALC) and peripheral blood mononuclear cells (PBMC) responses.

**Figure 1:**
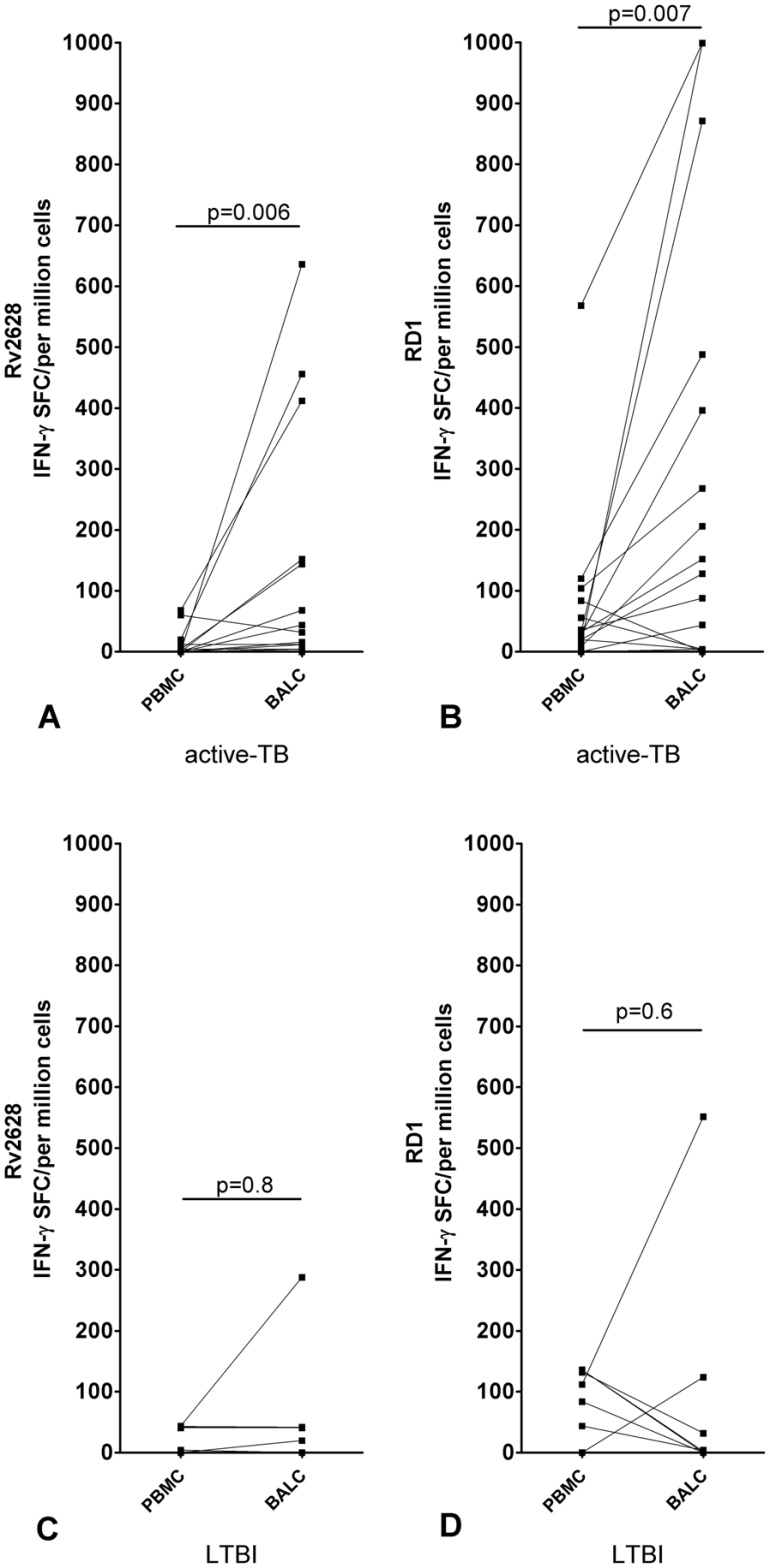
Increased frequency of Rv2628- and RD1-response in BALC than PBMC in active TB, evaluated by ELISPOT. ELISPOT evaluation of IFN-γ-producing CD4^+^ T-cells in circulating and BAL lymphocytes in response to Rv2628- and RD1-antigens in active TB and LTBI subjects. Response to Rv2628- (A) and RD1-antigens (B) in active TB patients; response to Rv2628- (C) and RD1-antigens (D) in LTBI subjects. **Footnote:** PBMC: peripheral blood mononuclear cells; BALC: bronchoalveolar lavage cells; SFC: spot-forming cells; IFN: interferon; TB: tuberculosis; LTBI: latent TB infection. Dotted lines link the results obtained for circulating and local lymphocytes for the same patient.

In the 16 patients with active TB who were tested, we observed a significantly higher number of IFN-γ-producing T-cells in response to both Rv2628 (BALC median: 24 spot forming cells (SFC)/10^6^ cells, interquartile range (IQR): 4–148 SFC/10^6^ cells; PBMC median: 0 SFC/10^6^ cells, IQR: 0–8 SFC/10^6^ cells) and RD1 antigens (BALC median: 140 SFC/10^6^ cells, IQR: 4–442 SFC/10^6^ cells; PBMC median: 30 SFC/10^6^ cells, IQR: 10–70 SFC/10^6^ cells) (p = 0.006 and p = 0.007, respectively) in BALC than in PBMC (Figure 1 A, B).

It was previously shown that compared to those with pulmonary TB, a low number of LTBI subjects had a sufficient number of BALC to perform a comparative analysis of both compartments. This is likely due to lower local cell activation and consequent recruitment in the lung [24]. Here we confirmed this data (table 2). Therefore, in the 7 subjects where the comparative analysis was possible, no significant difference was found in terms of IFN-γ-producing T-cells of BALC compared to PBMC in response to Rv2628- (BALC median: 41 SFC/10^6^ cells, IQR: 0–42 SFC/10^6^ cells; PBMC median: 41 SFC/10^6^ cells, IQR: 0–43 SFC/10^6^ cells) and RD1-antigens (BALC median: 4 SFC/10^6^ cells, IQR: 0–124 SFC/10^6^ cells; PBMC median: 112 SFC/10^6^ cells, IQR: 44–136 SFC/10^6^ cells), (p = 0.8 and p = 0.6, respectively) (Figure 1 C, D).

**Figure 2:**
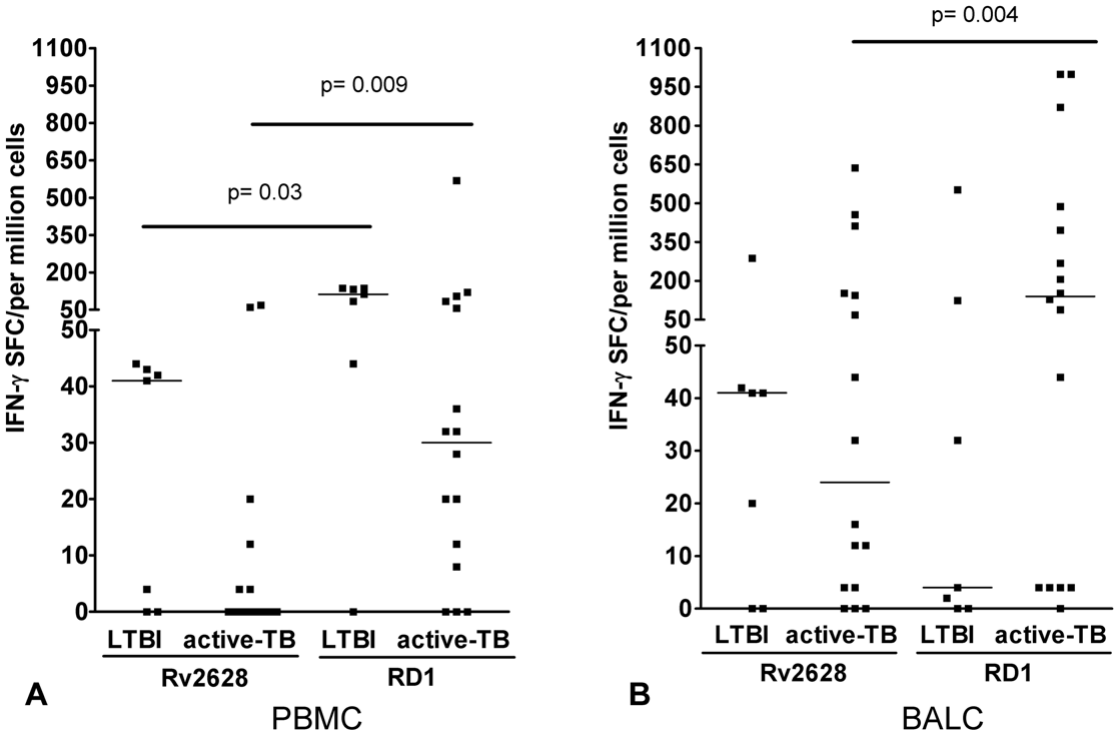
Magnitude of RD1-response is significantly higher than Rv2628-response in PBMC and BALC, evaluated by ELISPOT. ELISPOT evaluation of IFN-γ-producing CD4^+^ T-cells in PBMC and BALC in response to Rv2628- and RD1-antigens in LTBI and active TB subjects. Response to Rv2628- and RD1-antigens in PBMC of active TB and LTBI subjects (A), response to Rv2628- and RD1-antigens in BALC of LTBI and active TB subjects (B) **Footnote:** PBMC: peripheral blood mononuclear cells; BALC: bronchoalveolar lavage cells; SFC: spot-forming cells; IFN: interferon; TB: tuberculosis; LTBI: latent TB infection.

To better characterize the response to Rv2628- and RD1-antigens, we evaluated the magnitude of response to these antigens in PBMC and BALC separately. The trend of the response to Rv2628 in subjects with LTBI was higher than in active TB in both PB and BAL, although this difference was not significant. In PBMC, the RD1 frequency was significantly higher than Rv2628 in both active TB (p = 0.009) and LTBI subjects (p = 0.03) (Figure 2A). Differently, in BALC (Figure 2B) the frequency of RD1 response was significantly higher than Rv2628 only among those with active TB (p = 0.004).

As previously shown for the RD1-secreted antigens [23], [24], these results show a significantly higher frequency of *Mtb*-specific responses to Rv2628 at the site of TB disease than at the periphery in patients with active TB. The magnitude of the response to RD1 is significantly higher than what reported for the antigen of latency. Moreover, the trend of the response to Rv2628 in subjects with LTBI is higher than in active TB in both PB and BAL, although this difference is not significant.

### Comparison of Rv2628- and RD1-induced cytokine responses in PB and BAL by FACS analysis

We analyzed separately the response to Rv2628- and RD1-antigens in active TB and LTBI subjects in PB and BALC by FACS analysis.

**Figure 3:**
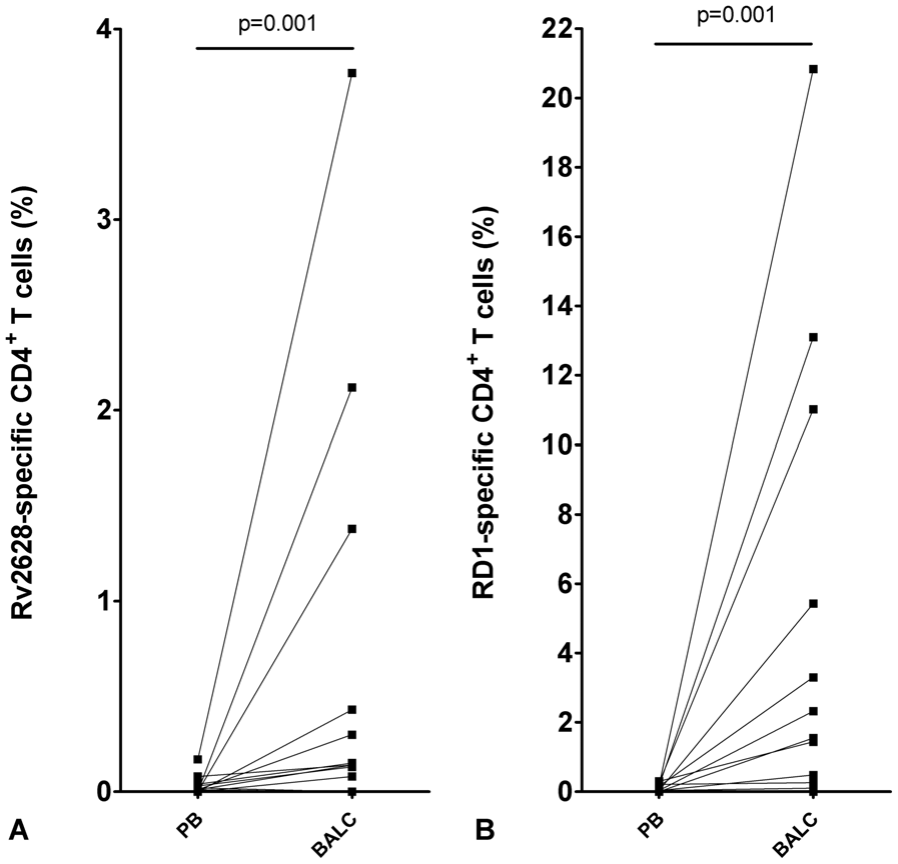
Increased frequency of Rv2628- and RD1-response in BALC than PB in active TB, evaluated by FACS. FACS analysis of the multiple cytokine producing CD4^+^ T-cells (IFN-γ and/or IL-2) in PB and BALC in response to Rv2628- (A) and RD1-antigens (B) stimulation in active TB patients. **Footnote:** PB: whole blood; BALC: bronchoalveolar lavage cells. Dotted lines link the results obtained for circulating and local lymphocytes for the same patient.

FACS analysis was performed simultaneously in 14 patients to evaluate T-cell multiple cytokine production (IFN-γ and/or IL-2). Among them, in line with results from the ELISPOT assay, we found a significantly higher number of multiple cytokine producing CD4^+^ T-cells in response to Rv2628 (BALC median: 0.14%, IQR: 0.04–0.90%; PB median: 0.01%, IQR: 0. –0.04%) and RD1antigens (BALC median: 1.49%, IQR: 0.05–8.22%; PB median: 0.04%, IQR: 0.01–0.19%) in BALC than in PB (p = 0.001 and p = 0.001, respectively) (Figure 3 A, B). Regarding the CD8^+^ T-cell specific response, we detected specific T-cell response to RD1 antigen in only one of the 14 samples evaluated, in both PB (2.31%) and BALC (8.50%).

Regarding the LTBI subjects, among the 5 subjects where sufficient cell numbers were available (table 2), no significant difference was found in the CD4^+^ T-cell response to Rv2628- and RD1-antigens (data not shown). No CD8^+^ T-cell response to Rv2628- and RD1-antigens was found (data not shown).

Altogether these FACS results confirm the data generated by ELISPOT.

### Cytokine profile in response to *Mtb*-specific antigens in active TB patients

To further assess the functional capacity of *Mtb-*specific T cells in active TB subjects, we analyzed the proportion of each cytokine subset contributing to the total RD1 and Rv2628 responses by FACS.

**Figure 4:**
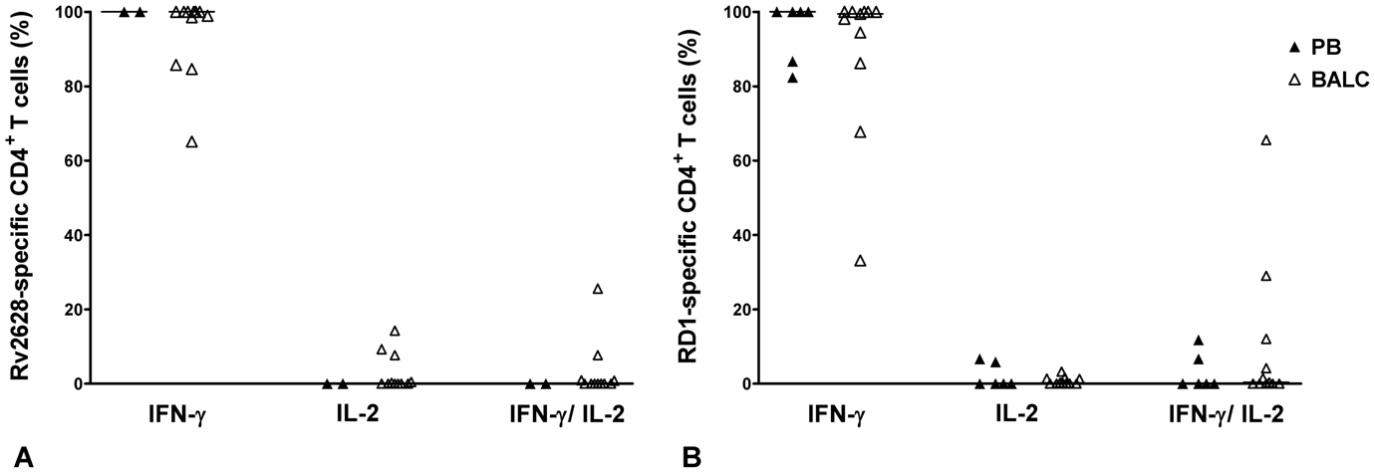
Cytokine profile in response to *Mtb*-specific antigens in active TB. Polyfunctional cytokine production analysis of Mtb-specific CD4^+^ T-cells by FACS. PB and BALC from active TB patients were stimulated overnight with Rv2628- and RD1-antigens. T- cells were classified as single IFN-γ-producing, single IL-2-producing or double IFN-γ/IL-2–producing cells in response to Rv2628- (A) and RD1-antigens (B). The results are reported as relative median in PB compared to BALC samples (A–B). Black triangles: PB; open triangles: BALC. **Footnote:** PB: peripheral blood; BALC: bronchoalveolar lavage cells; IFN: interferon; IL: interleukin.

Based on the cut-off (see MATERIALS and METHODS section), a robust analysis of cytokine profiles and CD4^+^ T-cell phenotypes in response to Rv2628 antigens was possible in 11 BAL samples and 2 PB samples of active TB patients. We found that the cytokine profile of response to Rv2628 was homogeneous and predominantly consisted of IFN-γ single-positive cells (PB median: 100%, IQR: 99–100%; BALC median: 100%, IQR: 85.71–100%). Interestingly, the single IL-2- and double IFN-γ/IL-2-producing cells were only present in BAL samples (IL-2 median: 0%, IQR: 0–7.69%; IFN-γ/IL-2 median: 0%, IQR: 0–0.94%) (Figure 4A).

Regarding the response to RD1-antigens, 11 BAL samples and 6 PB samples were further analyzed. We observed a homogeneous cytokine profile that predominantly consisted of IFN-γ single-positive cells (PB median: 100%, IQR: 85.59–100%; BALC median: 99.46%, IQR: 86.21–100%), with a smaller percentage of single IL-2-producing cells (PB median: 0%, IQR: 0–6.07%; BALC median: 0.18%, IQR: 0–1.39%), and double IFN-γ/IL-2-producing cells (PB median: 0%, IQR: 0–7.94%; BALC median: 0.36%, IQR: 0–12.07%) (Figure 4B).

**Figure 5:**
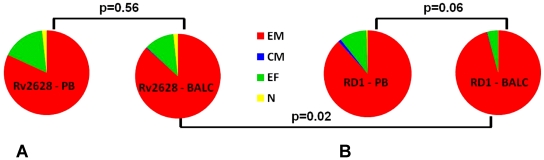
Phenotype profile in response to *Mtb*-specific antigens in active TB. Phenotypical T-cell subset distribution of the total cytokine-producing T-cells in response to Rv2628- (A) or RD1-antigens (B) are reported in PB and BALC samples. **Footnote:** PB: peripheral blood; BALC: bronchoalveolar lavage cells; N: naïve; CM: central memory; EM: effector memory; EF: terminally^-^differentiated effector memory.

Furthermore, we evaluated the phenotype of the total cytokine-producing cells. EM cells constituted the main phenotypic population in both PB (82%) and BAL samples (87%) in response to Rv2628 antigen (p = 0.56). Moreover as shown in figure 5A, there was only a small proportion of EF cells (11%) in BAL samples whereas other phenotypic populations were also represented in PB samples (16% EF, and 2% N).

Among the RD1 responders, the proportion of EM cells was almost significantly higher in BALC (96%) than in PB samples (88%) (p = 0.06). Moreover, there was only a small proportion of EF cells (4%) in BAL, whereas other phenotypic populations were also present in PB (10% EF, 1% CM and 1% N) (Figure 5B).

Comparing the response to the different antigens in BALC, a significantly higher number of EM cells responded to RD1 than to Rv2628 (p = 0.02) (Figure 5 A, B).

Based on the fact that only few LTBI patients showed a response to RD1 and Rv2628 (Figure 1C, D) in BAL, antigen-specific T-cell frequencies in LTBI patients were too low to comparatively assess cytokine profiles or T-cell phenotypes from PB and BALC in a robust manner. Therefore, no further FACS analysis was performed in this group.

Altogether these FACS results indicate that in patients with active TB, a higher proportion of EM cells respond to RD1-secreted antigens in BAL than in PB. In addition, in the BAL, a higher proportion of EM cells respond to RD1 than to Rv2628.

## Discussion

For the first time to our knowledge, we demonstrated compartment-related differences in the frequency and phenotypes of specific T-cell immunity for the *Mtb*-latency antigen Rv2628 in active TB patients who were prospectively enrolled with suspected active disease. Moreover, we also characterized the effector/memory phenotype of the BALC responding to RD1-secreted antigens, different from studies reported *ex vivo* or in pleural fluid [28]–[30].

In particular, in this observational study, we showed a higher frequency of CD4^+^ T-cells in BAL than in the PB of active TB patients for a latency antigen, not only for RD1 as previously shown [23]–[25]. Specifically, ELISPOT results, confirmed by FACS analysis, show that there was a significantly higher frequency of IFN-γ-producing antigen-specific CD4^+^ T-cells in BALC than in PBMC. The frequency of response to RD1 antigens was significantly higher than what was reported for Rv2628 in BAL and peripheral blood. Secondly, we showed that the cytokine profile predominantly consisted of IFN-γ single-positive cells over double IFN-γ/IL-2 positive T-cells, both in PB and BALC samples, independent of the stimulus. Consistent with previous observations [28]-[30], BAL-specific CD4^+^ T-cells presented predominantly an EM phenotype, while peripheral T-cell phenotypes were distributed among broader phenotypes (N, CM, EM and EF). Moreover, a significantly higher proportion of EM T-cells responded to RD1 than to Rv2628 in BAL. Finally, among the subjects with LTBI, the response to Rv2628 in BAL was not significantly higher than in PB, as previously shown for RD1 [23], [24]. Altogether our data contribute to a better understanding local immunity to *Mtb* in the lung.

We recently showed that the response to Rv2628-antigen in blood is associated with protection [14]. In agreement with this report, here we demonstrate that the trend of the response to this antigen of latency in subjects with LTBI is always higher than in active TB in both PB and BAL, although the difference is not significant. Interestingly, in active TB patients we found that the response to this antigen is also present at the site of infection and is significantly higher than in the periphery. These data are in agreement with those reported on HBHA [31]–[33], another latency antigen, which showed that the response was significantly higher in the lungs than in the periphery [34], where immune suppression to this antigen is mediated by T-regulatory cells [32]–[34].

Recent studies on patients with pleural TB showed that there is a higher proportion of pleural cells (PFMC) with an EM phenotype than other phenotypes in the periphery, both by *ex vivo* analysis and after specific stimulation [30]. Moreover, a higher proportion of poly-functional cells was found in PFMC than in PBMC after specific *in vitro* stimulation and had an EM rather than a CM phenotype [30]. On the contrary, in the present study we found predominantly mono-functional cells (only IFN-γ-producing CD4^+^ T-cells) in both BAL and PB samples from active TB patients. The reason for our different findings may be due to the different compartments analyzed (BALC vs PFMC).

IFN-γ-release assays on lymphocytes from BAL during mycobacterial infections have been suggested as being a potential new diagnostic tool for active TB [23], [25], [28]. In this study we confirm that RD1 responses in the BAL are associated with active disease. For the first time we show that the response to a latency antigen is increased in BAL than the periphery of patients with active TB. Nevertheless, at both PBMC and BAL levels, the response to Rv2628 tended to be higher in subjects with LTBI than in those with active disease, in line with our previous observations in whole blood [14]. Further studies are needed to evaluate the diagnostic potential of this result.

Both CD4^+^ and CD8^+^ T-cells with double and mono-functional response profiles to *Mtb* latency antigens can be detected with substantial frequency in long-term latently infected individuals [18]. In the present study only one patient with active TB showed CD8^+^ T-cell cytokine production in response to *Mtb-*specific stimulation. No CD8^+^ T-cell specific response was found in LTBI subjects. However, only few LTBI individuals were evaluated and they were not long-term infection controllers.

In this study we found that a large proportion of the specific T-cells are EM. This result is important in light of the reports performed in animal models. These reports show that EM T-cells, following antigen presentation in the local draining lymph nodes, migrate to the lung [35] and become involved in local host defense against pathogens through macrophage activation and neutrophil recruitment [28], [36].

There are some limitations in this study. We analyzed a relatively small number of subjects, especially for the LTBI group. Given the appreciable risk associated with bronchoscopy, our institute only provides this procedure for those individuals with a high suspicion of active TB and who result negative to AFB smear in the sputa. Although larger sample sizes with consequently longer time periods for enrolment would have probably increased the total number of individuals with LTBI, antigen-specific T-cell frequencies in BAL samples would likely be low in general [23], [24], and therefore preclude a large scale analysis of cytokine profiles and T-cell phenotypes in individuals with latent infection. Moreover, in active TB, both patients with microbiological and clinical diagnosis were included without distinct subgroup analysis. However, the results among the two subgroups were comparable (data not shown) and consequently combined. Despite these limitations, this study is unique because for the first time it provides information regarding the response to the antigens of latency at the site of *Mtb* infection, and their phenotypic characterization.

In summary, our results indicate that there is a high frequency of specific T-cells to both secreted (RD1) and latency-associated (Rv2628) antigens in the BAL of patients with active TB and that the majority of IFN-γ-only secreting T-specific cells in BALC have an EM phenotype. In light of the fact that not all lesions in the human lung are active during the disease [2], [4] and that consequently *Mtb* infection may be viewed as a continuous spectrum extending from sterilizing immunity to fulminant TB [2], [4] these data may be important for better understanding the pathogenesis of TB in the lung.

## Materials and Methods

### Ethics Statement

This study was approved by the Ethical Committee of our institution, the L. Spallanzani National Institute of Infectious diseases (INMI), approval number 70/2005. Informed written consent, required in order to participate in the study, was obtained.

### Patients

Patients admitted to INMI between 2009 and 2010 because of a clinical suspicion of TB were considered for enrollment in this study. According to the institutional protocol (http://www.inmi.it/protocolli) BAL is indicated for TB suspects if 3 expectorated or 2 induced-sputa result AFB smear negative and no alternative diagnosis is performed. TB suspects were enrolled in the study if: i) they underwent BAL as part of the confirmatory procedure for TB diagnosis, ii) they provided signed informed consent, iii) at least 6×10^6^ mononuclear cells from BAL could be recovered, iv) they tested negative for HIV and were not undergoing treatment with immunosuppressive drugs.

BAL was performed by instilling a sterile isotonic saline solution (4 times 30 ml) into an affected lung segment.

Active TB was defined as microbiologically confirmed if the BAL resulted *Mtb* culture-positive. Patients who resulted negative to *Mtb* culture in BAL were classified as having “clinical TB” when i) clinical, pathological and radiological findings consistent with TB were documented; ii) an alternative diagnosis was excluded, and iii) a full course of anti-TB treatment was started and an appropriate clinical/radiological response was obtained.

Patients with lung diseases other than TB had a final diagnosis made based on microbiological and cytological tests, clinical and radiological signs, and successful treatment.

### Blood and BAL Processing

Heparinized PB was collected and processed within 2 hours. PBMC were isolated by standard methods on Ficoll-Paque Plus (GE Healthcare Bio-Sciences AB, Uppsala, Sweden) [21]. BALC were obtained by passing the fluid through a sterile cell strainer with a pore size of 100 µm (BD Becton, Dickinson and Company, Milan, Italy) and then washing them with PBS.

### QuantiFERON TB-Gold In tube (QFT-IT)

QFT-IT (Cellestis Limited, Carnegie, Victoria, Australia) was performed. Results were scored as indicated by the manufacturer.

### IFN-γ ELISPOT

250,000 BALC or PBMC were stimulated with Rv2628 latency antigen at 10 µg/ml and RD1-antigen at 4 µg/ml or with the mitogen (positive control) (from the T-SPOT.TB, Oxford Immunotec Ltd., Abingdon, UK following the manufacturer’s instructions). Unstimulated sample (negative control) was included. ELISPOT results for Rv2628- and RD1-stimulations were scored as positive if more than 7 SFC/well were counted in the stimulated wells after subtracting the number of SFC in the negative control. ELISPOT results were scored as i) negative if they did not meet the definition for a positive result, ii) indeterminate if more than 10 SFC/well in the negative control were counted and iii) anergic if less than 20 SFC/well were counted in the positive control.

### Intra-cellular staining (ICS)

PB (100 µl) and BALC (10^6^) were stimulated overnight at 37°C in 5% CO_2_ with recombinant *Mtb*-specific antigens [37] identified as Rv2628 at 10 µg/ml and RD1 proteins (Lionex, Braunschweig, Germany) at 4 µg/ml, respectively. Phorbol-12-myristate-13-acetate (PMA) at 3 nM (SERVA Electrophoresis GmbH, Heidelberg, Germany) plus ionomycin at 1.5 µM (SERVA) was used as positive control. Cells were co-stimulated with anti-CD28 and anti-CD49d monoclonal antibodies (MoAb) at 2 µg/ml each (BD). Unstimulated PB and BALC were used to assess non-specific background cytokine production. Brefeldin A (SERVA) at 50 µg/ml was added after 1 hour of stimulation.

ICS was performed to measure IFN-γ and IL-2 production by CD4^+^ or CD8^+^ T-cells upon *in vitro* stimulation. PB and BALC were harvested, washed in PBS containing 1% BSA and 0.1% sodium azide (NaN_3_), and then stained with monoclonal antibodies (MoAb) directly conjugated to fluorochromes: allophycocyanin (APC)-H7-conjugated anti-CD4; peridinin chlorophyll-protein (PerCP)-Cy5.5-conjugated anti-CD8; phycoerythrin (PE)-Cy7-conjugated anti-CD45RO, and allophycocyanin (APC)-conjugated anti-CD62L (all from BD) in an incubation buffer (PBS-1% BSA-0.1% NaN_3_) for 30 min at 4°C. Subsequently, PB and BALC were washed, permeabilized with PBS-1% BSA-0.5% saponin-0.1% NaN_3_ and stained for 15 min at RT for intracellular cytokines with fluorescein isothiocyanate (FITC)-conjugated anti- IFN-γ and anti-IL-2-PE, or isotype-matched control MoAb (all from BD). Cells were washed, fixed in 2% paraformaldehyde, and at least 100,000 lymphocytes were acquired using a FACSCanto II flow cytometer (BD Biosciences), following gating according to forward and side scatter plots. FACS plots were analyzed using BD FACSDiva software (version 6.1.1). Frequencies of the different combinations of IFN-γ and/or IL-2 -positive cells following antigenic stimulation were calculated within the total population of CD4^+^ T-cells, and background values (unstimulated sample) were subtracted. Values corresponding to spontaneous IFN-γ and IL-2 production in the absence of *in vitro* stimulation were subtracted from the values obtained after antigen stimulation. At least 30,000 CD4^+^ T cells were analyzed. We established the lowest limit of detection for performing the analysis at 0.06% in order to have at least 18 events to analyze. FACS results were considered anergic if unresponsive to the positive control.

### Statistical analysis

Data were analyzed by the GraphPad Prism software, version 4.00 for Windows (GraphPad Software, San Diego, CA, USA). For continuous measures, medians and IQR were calculated. The significance of the differences between the two groups was determined using the non-parametric Mann-Whitney test or the Willcoxon test, when paired values were compared. Kruskall Wallis was used to compare medians among the different groups. For dichotomous measures, chi square was used. Differences were considered significant at p values ≤0.05.
